# Gnathic bone metastasis: a retrospective study of 10 cases

**DOI:** 10.1016/S1808-8694(15)30603-0

**Published:** 2015-10-18

**Authors:** Antonio Azoubel Antunes, Antonio Pessoa Antunes

**Affiliations:** 1 Dental surgeon, student in the specialization course in Buccomaxillofacial Surgery and Trauma.; 2 Head & neck surgeon. Oncology Center (CEON) of the Hospital Universitário Oswaldo Cruz (HUOC), Universidade de Pernambuco (UPE).

**Keywords:** mandible, maxilla, neoplasm metastasis, head and neck neoplasms, bone neoplasms

## Abstract

Oral cavity metastases are extremely rare, and comprise 1% of all mouth malignant neoplasms.

**Aim:**

to retrospectively analyze the incidence of maxillary metastases, and to trace its epidemiological behavior.

**Material and Methods:**

A retrospective study was done of the period between January 1980 and January 2000;the following indicators were reviewed: sex, age, site of the metastasis, site of the primary tumor, and the histological type of tumors in 10 patients.

**Results:**

The prevalence was similar in males and females (05 cases - 50%); three cases presented in the 2nd and 4th decades of life (30 - 30%). About half of the cases of metastases occurred in the maxilla and half in the mandible. The thyroid and prostate glands were the most prevalent primary tumor sites for metastases (03 cases each); the adenocarcinoma histological type had the highest metastatic rate (50%). Conclusions: Metastasis of the jaws is very rare and may occur at any age in both the genders, where the prevalence is equal for each sex. The thyroid and prostate seem to be the most frequent sites of distance metastasis of the jaws; the adenocarcinoma is the most frequent histological type causing such metastases.

## INTRODUCTION

Metastases in the mouth are extremely rare; they comprise 1% of all malignant mouth neoplasms. They are located in the mandible in 80 to 90% of cases; maxillary metastases are less common. Many types of primary malignancies can produce mouth metastases; the most of these tumors are located in the breast, the prostate, the lungs, the thyroid gland and the kidneys; these primary tumors give rise to most bone metastases.^1^

Metastatic involvement of the mandible and maxilla cause a variety of signs and symptoms. In some cases, patients may be asymptomatic, in which case the diagnosis is made as a radiological finding.

Mandibular and maxillary tumors cause significant morphological and functional changes, due to their natural progression and local/regional involvement; in most cases, a multidisciplinary approach is required.^2^

The differential diagnosis of these neoplasms may be difficult. Laboratory exams, such as immunohistochemistry, may provide some parameters depending on their accuracy. Paresthesia (unilateral, hypoesthesia or lower lip anesthesia), with or with no pain, may be the initial clinical manifestation in a significant percentage of mandibular bone lesions. Changes in sensitivity of the lower lip, even if there are no radiological findings, should alert specialists to think about the possibility of an initial stage metastasis.^3^

Bone metastases are relatively common findings in advanced carcinomas, especially those originating from primary breast, lung, thyroid, prostate and kidney tumors. In gnathic involvement, the mandible is the preferred site relative to the maxilla in a 4:1 proportion; its posterior portion is particularly involved, due to abundant vascular and hematopoietic tissue.^3^

The purpose of this study was to undertake a retrospective analysis of the incidence of maxillary metastases during a 20-year period in patients seen at a reference medical unit, and to investigate its epidemiological behavior based on the sample data.

## MATERIAL AND METHOD

We carried out a retrospective study from January of 1980 to December of 2000, comprising the patients seen and treated in the Head and Neck Surgery Ward of the Oncology Center - Centro de Oncologia (CEON) of the Oswaldo Cruz University Hospital (HUOC) - University of Pernambuco (UPE).

During this period, there were 190 recorded cases of head and neck bone tumors, of which ten patients (5.2%) had maxillary metastases. There were six female and four male patients aged between 13 and 75 years (mean - 43 years). The metastasis site, the primary tumor site and the histological tumor type were investigated (Table 1).

**Table 1:** Data of patients.

Patient	Gender	Age	Site	Site of primary tumor	Histological type
1	F	13	Maxilla	Thyroid	Papillary adenocarcinoma
2	F	64	Mandible	Thyroid	Follicular carcinoma
3	M	75	Mandible	Prostate	Adenocarcinoma NOS.
4	F	62	Mandible	Breast	Poorly-differentiated adenocarcinoma
5	F	75	Mandible	Thyroid	Follicular carcinomar
6	M	19	Maxilla	Soft part	Alveolar sarcoma
7	M	38	Maxilla	Prostate	Adenocarcinoma NOS
8	M	32	Maxilla	Prostate	Adenocarcinoma NOS
9	F	36	Maxilla	Breast	Ductal carcinoma
10	F	16	Mandible	Soft part	Myxoid fibrosarcoma

Key: NOS - not otherwise specified.

The Research Ethics Committee of the said institution registered and approved this study (protocol nº 134707/07).

**Figure 1:**
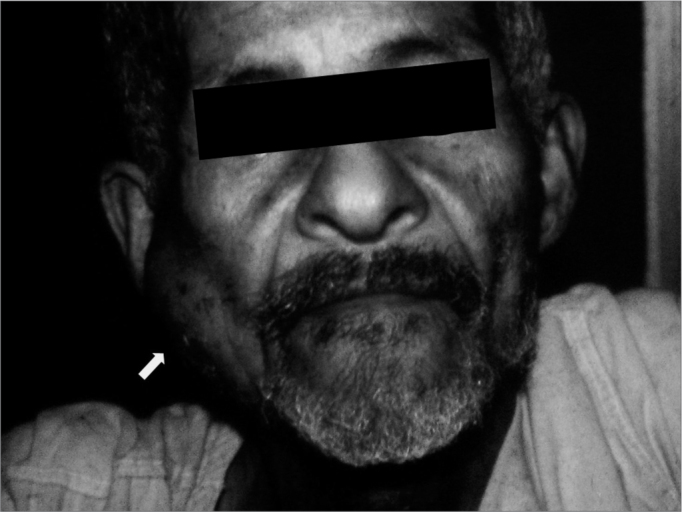
Clinical aspect of mandibular metastasis of a prostatic adenocarcinoma.

**Figure 2:**
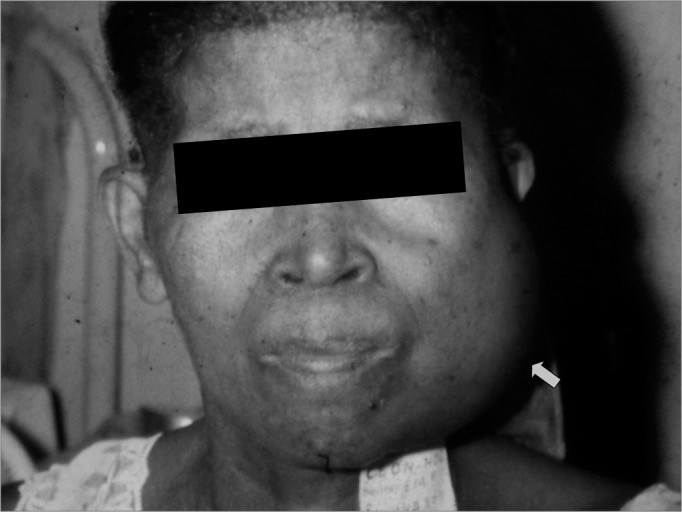
Clinical aspect of mandibular metastasis of a breast adenocarcinoma.

## RESULTS

The sample was as follows, based on our data:

## DISCUSSION

Malignant gnathic bone neoplasms, particularly of the mandible, have a low incidence compared to human tumors in general (less than 0.7%). These neoplasms cause significant morphological, functional and esthetic alterations due to their progression, local and regional involvement, and the need for aggressive therapy. They may be divided into primary and secondary or metastatic malignancies. There are three histological origins: tumors derived from odontogenic tissues, those derived from skeletogenic connective tissues, and those derived from non-odontogenic and non-skeletogenic tissues.^2^

Gnathic bones are generally uncommon sites for metastases; their involvement may, however, be higher than the usually reported frequency in the literature.^4^

Rutsatz et al.^5^ (1990) reported 1,008 patients with malignancies of the face (soft parts and bone) and mouth in a 35-year period, and found only five patients (0.5%) with metastases to the mandible. These were described as distance metastases of tumors from various primary sites.

Pruckmayer et al.^6^ undertook a major retrospective study of 763 patients that presented non-specific pain in the mandible; of these, only nine patients (1.2%) had metastases to the mandible. These authors noted that pain was an important sign in the investigation, and that gnathic bone metastases indicate widespread disease,^4^, ^6^ as seen in our sample.

There were three cases in the second and fourth decades of life (30%), and two cases in the seventh and eighth decades of life (20%). The age ranged from 13 to 75 years (median - 43 years).

Van der Waal et al.^7^ analyzed a sample of 1,537 mouth cancer cases, of which 24 were metastatic tumors; the age distribution was similar to that of our sample, ranging from 8 to 90 years (median - 60 years).

D'Silva et al.^8^ commented the age and sex distribution of maxillary metastases in their sample, stating that females have twice the number of metastases compared to males. The age groups in that study, from higher to lower occurrence, were 31 to 40 years and 71 to 80 years, different from our findings.

The mandible and the maxilla were equally involved by bone metastases (5 cases each), which is different from data in the literature, which indicates an 80% prevalence of bone metastases in the mandible.^4^, ^9^

D'Silva et al.^8^ studied 114 cases of maxillary metastases retrospectively, and reported that the preference for the mandibular bone was more evident in females, rather than males.

Hirshberg et al.^10^ studied 390 cases of gnathic bone metastases, of which 316 cases (81%) were located in the mandible, and only 58 cases (14%) were located in the maxilla; involvement of both bones was seen in 21 patients (5.4%).

The metastatic tumor histology in our sample revealed papillary adenocarcinoma (one case), poorly-differentiated adenocarcinoma (one case), adenocarcinoma not otherwise specified (three cases), follicular carcinoma (two cases), myxoid fibrosarcoma (one case), alveolar sarcoma (one case) and ductal carcinoma (one case)

Van der Waal et al.^7^ have stated that the adenocarcinoma is the main histological type of gnathic bone metastases from a variety of primary tumors, which is in agreement with the data from our sample.

In our sample, the sites of primary malignancies giving rise to distance metastases to gnathic bones were the thyroid gland (3 cases) and the prostate (3 cases) as the three most common sites, followed by the breast (2 cases) and soft part sarcomas (2 cases).

Soft part metastases to the mouth are uncommon, but of significant occurrence. The literature cites the breast, the thyroid and the lung as the most common sites responsible for hematogenic distance metastases to gnathic bones.^3^, ^11^, ^12^ Other sites, such as the prostate, soft parts and the kidneys, are considered as rare;^3^ still other sites mentioned below are extremely rare.

Piattelli et al.^13^ investigated 390 mouth cancer cases and found 22 cases (5.6%) of gnathic bone metastases, of which the prostate was the primary site. These authors underline that the prostate is a rare site for primary malignancies giving rise to maxillary metastases.

Stavropoulos and Ord^14^ reported a case of a female patient with breast cancer that metastasized to the mandibular condyle; there ensued a pathological fracture that was initially diagnosed and treated as a non-neoplastic manifestation of the temporo-mandibular joint.

Porter et al.^15^ reported a case of malignant teratomas of the testicle that metastasized to both mandibular condyles. The diagnosis was only made because the patient complained of intense pain in the affected sites. Schumacher and Rohde^16^ reported a rare case of vulvar carcinoma that metastasized to the mandible. Yanagi et al.^11^ reported a case of a male patient aged 59 years with a malignant pheocromocytoma - a primary tumor o the right adrenal gland - that metastasized to the mandible. Ogunsalu and Samith^17^ reported a case of a right adrenal neuroblastoma in a male patient aged 21 years that metastasized to the mandible. Rare sites, such as soft parts (sarcomas) and primary tumors from other bones rarely metastasize to the mandible and maxilla.^4^

There are cases of neck soft part neoplasms that grow by extension into adjacent structures; these tumors may simulate a primary tumor in radiological exams. Captier et al.^18^ reported a case of a child aged 10 years that presented a tumor on the horizontal ramus of the mandible that extended to the submandibular area. Radiology suggested a primary bone neoplasm, but histopathology revealed a soft part synovial sarcoma of the neck that invaded the mandible and the floor of the mouth; this is an extremely rare mesenchymal tumor of the head in children.

The literature states that in about 30% of cases of patients with gnathic bone metastases, the primary tumor is asymptomatic and not diagnosed.^9^ No hidden primary tumors with existing metastases were observed in our sample.

The prognosis of gnathic bone metastatic neoplasms is poor, resulting from the pathophysiology of the primary tumor, which generally is highly aggressive; these metastases occur at an advanced stage of the disease, and may be associated with paraneoplastic syndromes.

## CONCLUSION

1. Gnathic bone metastases are rare and may affect any age group and both sexes, with equal prevalence in the mandible and maxilla.
2. The thyroid gland and the prostate appear to be the most frequent primary sites for distance metastases to gnathic bones. The adenocarcinoma appears to be the most common histological type in metastases.
